# Supplementary material for: In silico identification of archaeal DNA-binding proteins
Source: Bioinformatics. 2025 May 2;41(5):btaf169. doi: 10.1093/bioinformatics/btaf169 (PMC12065626; doi:10.1093/bioinformatics/btaf169)
Supplement: btaf169_Supplementary_Data [file btaf169_supplementary_data.pdf]

In-silico identification of archaeal DNA-binding proteins

Linus Donvil [1], Joëlle A.J Housmans [4,5], Eveline Peeters [1], Wim Vranken[2,3] and Gabriele Orlando[4,5]

[1] Research Group of Microbiology, Department of Bioengineering Sciences, Vrije Universiteit Brussel, Pleinlaan 2, B-1050 Brussels, Belgium
[2] Interuniversity Institute of Bioinformatics in Brussels, ULB/VUB, Triomflaan, 1050 Brussels, Belgium
[3] Structural Biology Brussels, Vrije Universiteit Brussel, Brussels 1050, Belgium
[4] Switch Laboratory, Department of Cellular and Molecular Medicine, KU Leuven, Leuven, Belgium
[5] VIB Center for Brain and Disease Research, Leuven, Belgium

SUPPLEMENTARY TABLES

Supplementary Table 1: Predicted Asgard proteins with high confidence as DNA-binding proteins. *The table lists the top 10 proteins from the Asgard group, ranked by Xenusia's highest confidence scores for their predicted DNA interactions. ABC, ATP-binding cassette; HTH, helix-turn-helix.*

#	Xenusia Score	Uniparc ID	Annotation
1	0.957	OLS30781	Metalloprotease (homology)
2	0.954	OLS30429	Characterized HTH
3	0.950	OLS21705	Characterized HTH
4	0.948	OLS27061	ABC transporter (homology)
5	0.947	OLS17998	HTH (homology)
6	0.947	OLS16705	HTH (homology)
7	0.945	OLS19303	Response regulator SaeR
8	0.945	OLS28978	Aminopeptidase BapA (homology)
9	0.943	OLS30097	-
10	0.941	OLS18037	S60 ribosomal protein (homology)

Supplementary Table 2: Three experimentally investigated Asgard proteins. *The table presents the three Asgard proteins that underwent experimental examination, along with their distant homologous transcription factors. The homologous protein and the sequence identity has been identified using the BLAST web server (Johnson et al. 2008).*

Xenusia Score	Uniparc ID	Homologous Organism	Sequence Identity
0.904	OLS17986	Sulfurisphaera tokodaii (archaea)	41%
0.926	OLS23561	Bacillus subtilis (bacteria)	43%
0.895	OLS31011	Haloferax volcanii (archaea)	43%

Supplementary Table 3: *List of dsDNA probes used in the DNA-binding assay.*

**Name**	**Sequence (5' - 3')**
*OLS17986*_Probe1f	tgttattcaaatgtgtgaacctctagtttcgagaactagtattacgtgatgttatgtttacctatttaaactttctgatttggtaaaaaaagttttaaaa
*OLS17986*_Probe1r	ttttaaaactttttttaccaaatcagaaagtttaaataggtaaacataacatcacgtaatactagttctcgaaactagaggttcacacatttgaataaca
*OLS17986*_Probe2f	attagcatattagctcaccttgaccttgtgagatttatattttttaaatatcactgaataataactaactaatacttaaatttaaaccttccagtattct
*OLS17986*_Probe2r	agaatactggaaggtttaaatttaagtattagttagttattattcagtgatatttaaaaaatataaatctcacaaggtcaaggtgagctaatatgctaat
*OLS17986*_Probe3f	aacataatttttctaacatccatcagggagtccacctagcaaagttaaaatgctagtcgattatactaacttttatttaacattaattagaacgcggtga
*OLS17986*_Probe3r	tcaccgcgttctaattaatgttaaataaaagttagtataatcgactagcattttaactttgctaggtggactccctgatggatgttagaaaaattatgtt
*OLS31011*_Probe1f	tcaatcaagagattcattctcccaagtaaataggtagattgaagtaggaatagtgttttgccgtcatgcgacgggtgtttataagatgatagaagctaaa
*OLS31011*_Probe1r	tttagcttctatcatcttataaacacccgtcgcatgacggcaaaacactattcctacttcaatctacctatttacttgggagaatgaatctcttgattga
*OLS31011*_Probe2f	gaatatctctgacaatcatggataacagtgaaatagcattgtgtcatgggtacaatgattacgatggtggattcggagtacttacaggataagaaagttc
*OLS31011*_Probe2r	gaactttcttatcctgtaagtactccgaatccaccatcgtaatcattgtacccatgacacaatgctatttcactgttatccatgattgtcagagatattc
*OLS31011*_Probe3f	ggttgaggatgaaaagaaaagagagattggacttcgactaacgaattcaatgggaggcttgaggagtgaacgcatatttcacaatagtactacttggatt
*OLS31011*_Probe3r	aatccaagtagtactattgtgaaatatgcgttcactcctcaagcctcccattgaattcgttagtcgaagtccaatctctcttttcttttcatcctcaacc
*OLS23561*_Probe1f	cacatatatacttttatagtaatattggttacaaatgttttcacctaattaaaaaacaattaataatgatatatttcgaggaatacctatgagagcaat

Name	Sequence (5' - 3')
*OLS23561*_Probe1r	attgctctcataggtattcctcgaaatatatcattattaattgtttttaattaggtgaaaacatttgtaaccaatattactataaaaagtatatatgtg
*OLS23561*_Probe2f	aatattcatttaaccacatggttgaatataatttagtgtatataaatatttcattcaactaaatggtataatgtttcttatccaatcaaataccaatatc
*OLS23561*_Probe2r	gatattggtatttgattggataagaaacattataccatttagttgaatgaaatatttatatacactaaattatattcaaccatgtggttaaatgaatatt
*OLS23561*_Probe3f	caaaaaccatatctcaattctcttaaattacttgcattttgcaagagaatcttattctgcatattcttaactaattattaatttggttcttaacttatcg
*OLS23561*_Probe3r	cgataagttaagaaccaaattaataattagttaagaatatgcagaataagattctcttgcaaaatgcaagtaatttaagagaattgagatatggtttttg

SUPPLEMENTARY FIGURES

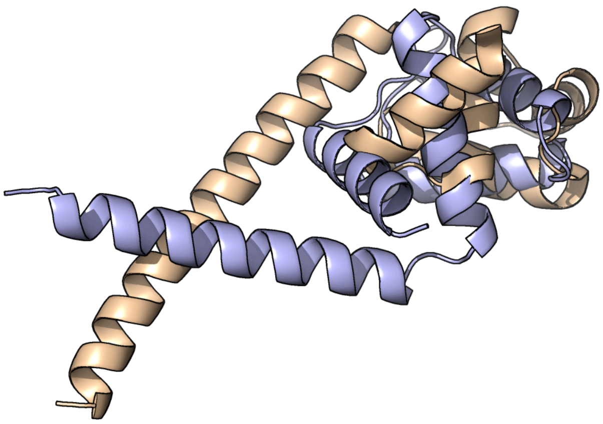

Supplementary Figure 1: Alignment of the AlphaFold predicted structure of an unannotated Asgard protein and the crystal structure of an archaeal HTH DNA-binding protein. *The image shows the structural alignment (performed with PyMOL) of the two protein structures shown in Figure 2. While the structure is similar, their sequence is reversed and the algorithm fails to align them.*

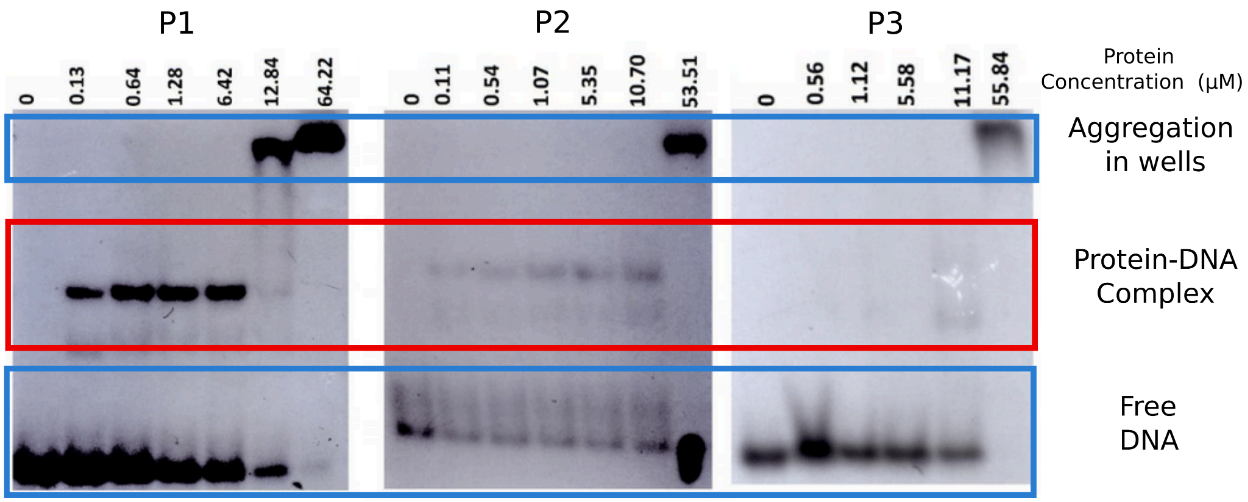

Supplementary Figure 2: DNA binding assay for OLS23561 using different protein concentrations. *The image shows the results of the DNA binding assay for OLS23561 at varying protein concentrations, addressing the aggregation problems observed in Figure 3. At low concentrations (<10 µM), no protein aggregation is observed. The red box indicates where the band of the monomeric protein-DNA complex should appear. The test reveals that the protein binds specifically to probes P1 and P2 but not to P3.*
